# Three Decades of Evidence on Pulsatile Cardiopulmonary Bypass in Pediatrics: No Significant Clinical Benefit

**DOI:** 10.1177/21501351251412887

**Published:** 2026-02-04

**Authors:** Akif Ündar, Marc J. Lussier, Srihari Rajesh, Kevsergül Dayi

**Affiliations:** 1 Penn State Hershey Pediatric Cardiovascular Research Center, 12310 Penn State College of Medicine, Hershey, PA, USA; 2 Division of Pediatric Cardiology, Department of Pediatrics, 12310 Penn State College of Medicine, Hershey, PA, USA; 3 Department of Surgery, 12310 Penn State College of Medicine, Hershey, PA, USA; 4 Department of Biomedical Engineering, College of Engineering, The Pennsylvania State University, University Park, PA, USA

**Keywords:** pulsatile and nonpulsatile flow, cardiopulmonary bypass, congenital heart surgery, vital organ injury, clinical outcomes, CPB circuits, pediatrics, neonates and infants

## Abstract

**Background:**

Drawing on more than 30 years of valuable expertise in in-vitro, in-vivo, and clinical trials, we report the key findings of our institutional research on pulsatile cardiopulmonary bypass (CPB).

**Methods:**

A detailed search was performed in the National Institutes of Health National Library of Medicine, focusing on the PubMed database. This search combined key terms such as “Pulsatile flow,” “Cardiopulmonary Bypass,” “Undar A,” “Vital Organ Injury,” “Clinical outcomes,” “Pediatrics,” and “Neonates and infants” to find relevant peer-reviewed publications.

**Results:**

A comprehensive review article was crafted by analyzing 88 peer-reviewed studies published between 1996 and 2025. This research provided substantial scientific evidence on optimizing neonatal and pediatric cardiopulmonary bypass (CPB) circuitry, focusing not only on pump performance but also on selecting appropriate oxygenators and arterial cannulas for effective pulsatile flow. While significant benefits regarding cerebral hemodynamics were observed in neonatal piglet models using pulsatile flow in conjunction with deep hypothermic circulatory arrest, the results from randomized clinical trials and retrospective studies raised questions regarding its efficacy. Despite some promising indications of improved Apolipoprotein E and plasminogen activator inhibitor-1/tissue plasminogen activator ratios, a rigorous randomized clinical trial involving 159 patients, along with a comprehensive retrospective study of 284 patients at Penn State Health Children's Hospital, demonstrated no significant clinical advantage of pulsatile flow compared with nonpulsatile flow. Importantly, while pulsatile flow did not provide a measurable benefit, it also did not result in any adverse outcomes in our clinical trials.

**Conclusions:**

The existing evidence clearly indicates that pulsatile CPB does not provide significant short-term or long-term benefits for pediatric patients undergoing congenital heart surgery.

## Introduction

The ongoing conversation about the effectiveness of different perfusion modalities in fostering vital organ recovery and enhancing clinical outcomes during cardiopulmonary bypass (CPB) procedures for patients with congenital heart conditions has been evolving for over 70 years. This enduring dialogue highlights not only the complexities involved but also the deep commitment within the field to prioritize patient well-being. Continued research and collaboration through a multidisciplinary team are essential to address the challenges patients face and to achieve a deeper understanding of their profound impact on patient care and recovery.

With more than three decades of dedicated expertise encompassing in-vitro, in-vivo, and clinical trials, we present the crucial findings of pulsatile CPB from our institutional research.^1–8^ Our mission is to develop compassionate and innovative solutions that address the underlying causes of these challenges, steering us toward impactful and constructive resolutions in this long-standing debate.^9^ Specifically, we aim to share robust, evidence-based strategies that significantly enhance patient safety for congenital heart surgery patients when pulsatile flow is preferred.^10^ This review article compiles the findings from our years of extensive research on the impact of pulsatile and nonpulsatile flow for pediatric congenital heart surgery patients. It is our hope that the insights gained from our studies shine a light on the profound relevance of this work and resonate with fellow researchers committed to advancing pulsatile CPB techniques for our most vulnerable patients—neonates and children facing congenital heart challenges.

## Patients and Methods

For the clinical portion of this review article, patients were enrolled at Penn State Health Children's Hospital only after obtaining parental consent. The IRB study ID for this research is PRAMS019299-A (last approval date July 14, 2025). This study is registered with the National Institutes of Health National Clinical Trial database (NCT00862407). The participants included in this study were all under 18 years of age and were undergoing CPB for congenital heart disease (CHD). Patients were randomly assigned to receive either pulsatile or nonpulsatile flow during the procedure. Moreover, we implemented a complementary clinical protocol aimed at intraoperative multimodality neuromonitoring during CPB. This protocol received Institutional Review Board approval (PRAMS030476EP) on February 5, 2025, and it significantly fortifies our manuscript by enabling the inclusion of data from the retrospective chart review.

**Figure 1.**
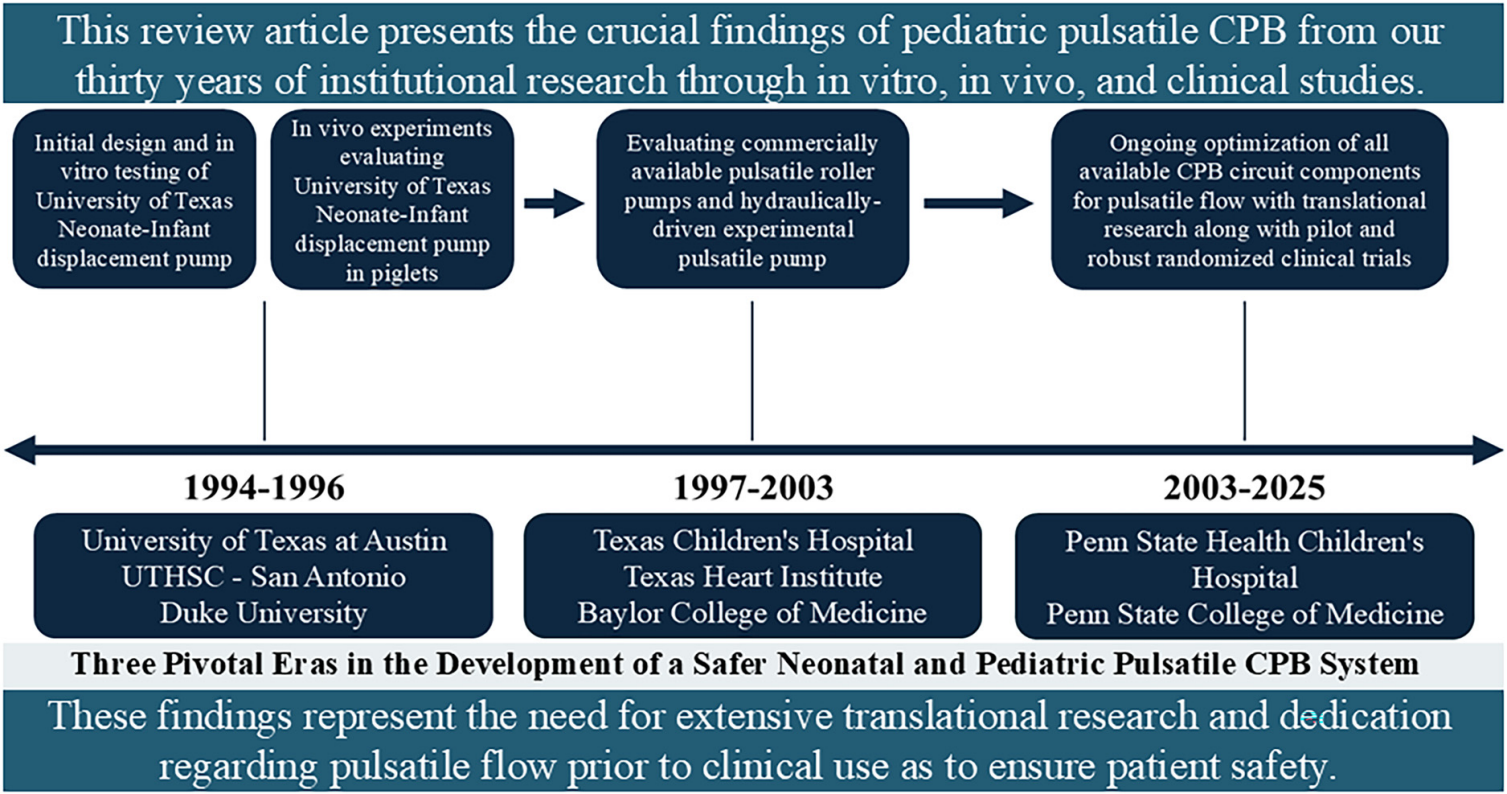
Three pivotal eras in the development of a safer neonatal and pediatric pulsatile cardiopulmonary bypass (CPB) system.

On July 18, 2025, an advanced search was conducted in the National Institues of Health National Library of Medicine, specifically within the PubMed database. The search utilized a combination of key terms, including “Pulsatile flow,” “Cardiopulmonary Bypass,” “Undar A,” “Vital Organ Injury,” “Clinical outcomes,” “Penn State College of Medicine,” “Pediatrics,” and “Neonates and infants,” to gather relevant peer-reviewed publications. The following description and Figure 1 outlines three pivotal eras in the development of innovative neonatal-infant and pediatric pulsatile CPB systems:

1. *Years 1994-1996*: This period marked the foundation of research at the Biomedical Engineering Program of the University of Texas at Austin, where initial experiments led to the design of an experimental neonatal-infant physiologic pulsatile CPB system using the University of Texas Neonate-Infant displacement pump. Pilot studies were conducted with neonatal piglets at the animal facilities of the Department of Surgery at the University of Texas Health Science Center in San Antonio, Texas. Then, comprehensive evaluations of the entire pulsatile flow circuit were performed, comparing its effectiveness against conventional nonpulsatile flow through a series of piglet experiments. These studies also included assessments of deep hypothermic circulatory arrest (DHCA) models and evaluations of membrane oxygenators in vivo at the Congenital Heart Surgery Laboratory at Duke University Medical Center in Durham, North Carolina.
2. *Era of 1997-2003*: Building upon early findings, this era focused on a thorough evaluation of all commercially available pediatric pulsatile roller pumps, alongside an innovative hydraulically driven experimental pulsatile pump specifically designed for neonates. These critical experiments were carried out with neonatal piglets at the Texas Children's Hospital, Baylor College of Medicine, and Texas Heart Institute, striving to enhance the safety and efficacy of pulsatile CPB systems.
3. *Era of 2003-2025*: In this ongoing phase, the focus has turned to the comprehensive optimization of all clinically available CPB circuit components. This includes the execution of pioneering pilot clinical trials and a robust randomized clinical trial exploring various perfusion modalities at the Penn State Health Children's Hospital and Penn State College of Medicine in Hershey, Pennsylvania.

## Results

A total of 88 articles emerged when exploring the terms “pulsatile flow,” “cardiopulmonary bypass,” and “Undar A.” Narrowing the focus to pediatrics, the search produced 56 relevant articles. Furthermore, by incorporating “neonates and infants” into the search, 35 studies were identified. The inclusion of “Penn State College of Medicine” brought forth 29 articles, while 17 results concentrated on “clinical outcomes.” (Supplemental Tables 1-3). This research highlights the substantial body of work in this vital area, particularly from institutional contributions.

### A) Design of a University of Texas Neonate/Infant CPB System: A Pioneering Journey (1994-1996)

#### Initial Conception and Optimization

In a series of extensive evaluations, both in vitro and in vivo, we aimed to demonstrate the remarkable capabilities of the University of Texas Neonate/Infant CPB system.^1,11–15^ Through careful measurements of pulse pressure, dp/dt, changes in pressure, stroke volume, ejection time, and flow rate, we found that this innovative pump could produce a physiological blood flow waveform (Supplemental Figure 1). By adjusting motor speeds, we were able to achieve varying physiological pump rates, flows, and stroke volumes, showcasing the system's adaptability.^1,11,12^

The physiologic pulsatile pressure-flow waveforms generated by this pump exhibit a quality that closely resembles natural pulsatility, as depicted in Supplemental Figure 1.^1,11^ It's important to note that while this pump is able to replicate a physiological quality of pulsatility, it does not produce conventional nonpulsatile flow.

We then paired the University of Texas Neonate/Infant CPB pump with various oxygenators and arterial cannulas to optimize pulsatile flow in three groups of neonatal piglets at Duke University.^13^ The Capiox 308 hollow fiber membrane oxygenator, paired with a 10F Elecath aortic cannula, consistently demonstrated the most effective physiological pulse pressure.^13–15^ In stark contrast, the VPCML Plus flat sheet membrane oxygenator failed to achieve physiological pulsatility, significantly dampening the pulsatile waveform.^13,14^ Hollow-fiber membrane oxygenators exhibit notably lower pressure drops, making them superior candidates for use with pulsatile pumps compared to their flat-sheet counterparts. However, it's crucial to note that the Minimax Plus, a hollow fiber option, has been linked to excessive blood trauma during hypothermic pulsatile CPB, underscoring the vital need for in vivo testing of CPB components with pulsatile flow.^13^

Furthermore, the specific brand of arterial cannula used has a direct and profound impact on the quality of pulsatility.^13^ This reinforces the necessity of meticulously selecting circuit components to maximize the physiological advantages of pulsatile flow. Alarmingly, the U.S. Food and Drug Administration (FDA) has yet to approve any components of the CPB circuitry designed for pulsatile flow in clinical practice, and currently, manufacturers have not provided any data evaluating membrane oxygenators in conjunction with pulsatile flow, particularly for congenital heart surgery patients.^9,10^ Therefore, conducting translational research is not merely beneficial but essential for ensuring the safety of CPB patients when pulsatile flow is prioritized in clinical practice.^10^

#### Regional and Global Cerebral Blood Flow Measurements Using the Radiolabeled Microsphere Technique

Following the instrumentation of 3 kg piglets for CPB, they were subjected to either nonpulsatile flow with a conventional roller pump or pulsatile flow utilizing the alternate University of Texas Neonate-Infant Physiologic pulsatile pump.^1,4,15^ Regional cerebral blood flow was meticulously assessed in the cerebellum, basal ganglia, brain stem, and both left and right cerebral hemispheres through the radiolabeled microsphere method. This involved injecting uniquely labeled 15-micron microspheres via the left atrial catheter before and after CPB, as well as into the aortic cannula during CPB. Measurements of regional and global cerebral blood flow were taken at a cerebral perfusion pressure (CPP) of 55 mm Hg before and after DHCA, as well as at 40 and 70 mm Hg CPP post-DHCA.^1,4^

**Figure 2.**
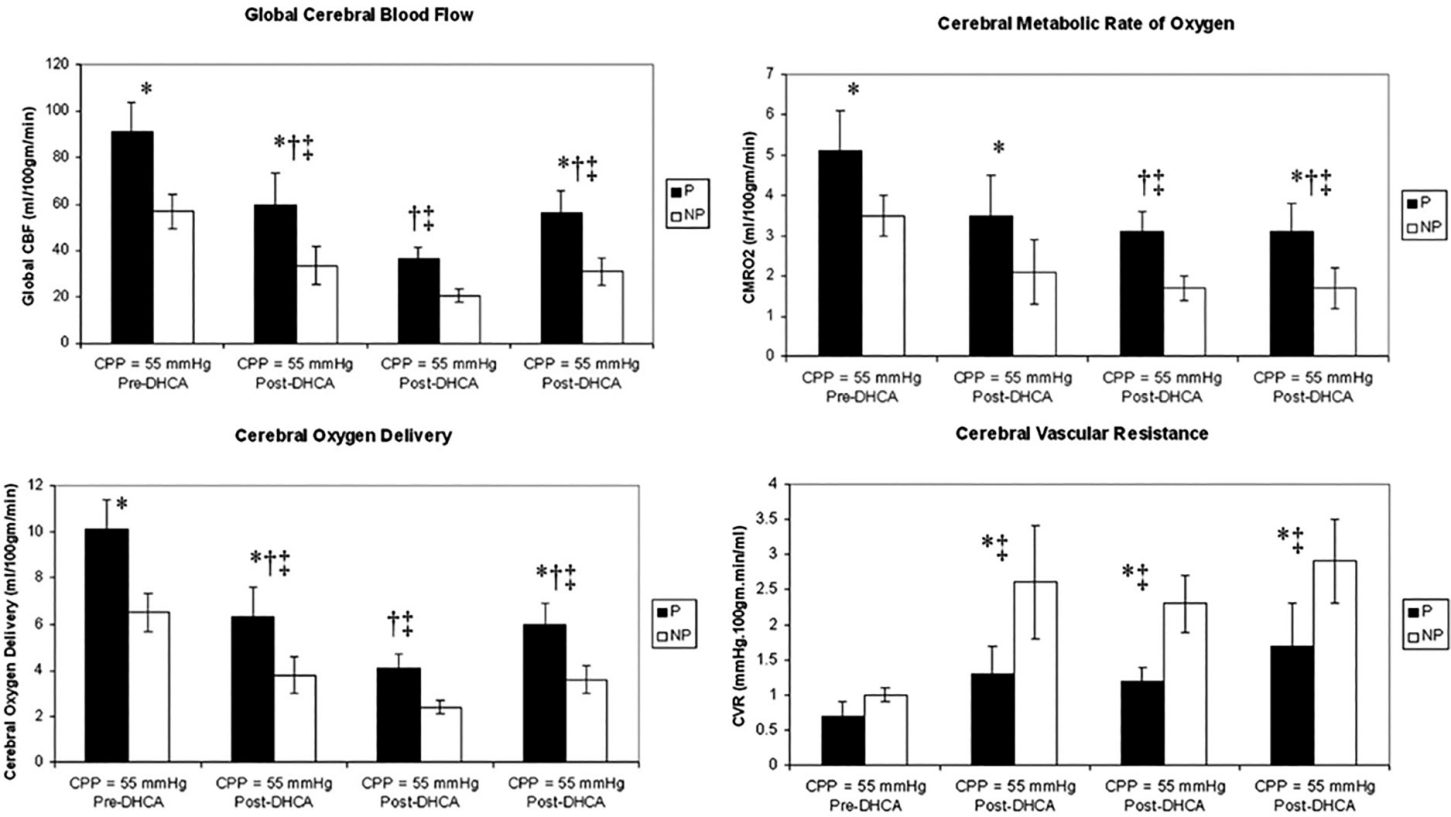
Cerebral hemodynamics in neonatal piglets undergoing cardiopulmonary bypass (CPB). Global cerebral blood flow, cerebral metabolic rate of oxygen, cerebral oxygen delivery, and cerebral vascular resistance during pulsatile versus nonpulsatile perfusion (mean ± SEM). *P < .05 P vs NP, †*P* < .05 versus pre-DHCA at CPP = 55 mm Hg within pulsatile group; ‡*P* < .05 versus pre-DHCA at CPP = 55 mm Hg within nonpulsatile group. CBF, cerebral blood flow; CPP, cerebral perfusion pressure; DHCA, deep hypothermic circulatory arrest; NP, nonpulsatile; P, pulsatile. [Reproduced with Permission from Ündar A, et al *ASAIO J*. 2005;51:vi-x.].

The findings demonstrated that the University of Texas Neonate/Infant CPB pump significantly outperformed the nonpulsatile conventional roller pump, yielding superior regional blood flows across all measured cerebral regions, including the cerebellum, basal ganglia, brain stem, and both hemispheres.^1,4^ Additionally, it achieved higher global cerebral blood flow, an increased cerebral metabolic rate of oxygen, and enhanced cerebral oxygen delivery at every experimental stage (Figure 2). Nonpulsatile flow, however, not only failed to deliver comparable benefits but also notably increased cerebral vascular resistance.^1,4^ These compelling results underscore the critical advantages of utilizing the physiological pulsatile pump to enhance cerebral perfusion during CPB procedures in neonatal piglets. In stark contrast, a separate study conducted under different experimental conditions revealed that in both pulsatile and nonpulsatile perfusion groups, while regional cerebral blood flow and viscoelasticity decreased after 60 min of DHCA, these metrics failed to return to pre-CPB levels even after an additional 60 min post-CPB.^16^ An additional study showed that although the University of Texas Neonate/Infant CPB pump consistently maintained myocardial blood flow at or above baseline levels throughout its duration, there were no significant differences in cerebral blood flow.^15^

### B) Experiments at Texas Children’s Hospital/BCM/THI (1997-2003)

#### Precise Quantification of Pressure Flow Waveforms

To effectively analyze and compare pulsatile versus nonpulsatile perfusion, precise quantification of pressure and flow waveforms is essential. The generation of pulsatile flow is driven by energy gradients rather than pressure gradients alone. To enrich our understanding, we have utilized Shepard's energy equivalent pressure (EEP) formula in our previous in-vitro and in-vivo studies focused on optimizing CPB circuits.^3,17,18^ This approach has enabled us to gain valuable insights and improve our methodologies. Since EEP is measured in mm Hg, it allows for a direct comparison with mean arterial pressure (MAP). Under pulsatile flow, EEP consistently exceeds MAP, and the disparity between the two represents the surplus hemodynamic energy (SHE).^19^

**Figure 3.**
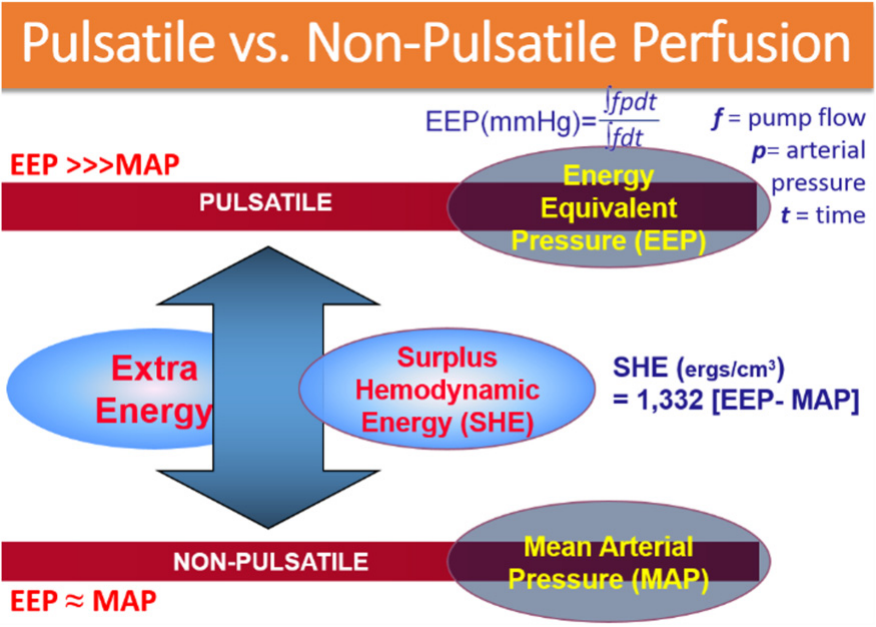
Diagram of energy equivalent pressure and surplus hemodynamic energy (SHE). Under identical experimental conditions, pulsatile flow significantly generates a greater amount of hemodynamic energy compared with nonpulsatile flow. Notably, both energy equivalent pressure (EEP) and SHE are consistently elevated with pulsatile flow. In fact, SHE reaches zero under conditions of 100% nonpulsatile flow, while EEP aligns directly with mean arterial pressure. This Diagram underscores the superior efficiency and benefits of pulsatile flow in enhancing hemodynamic performance.

In identical experimental settings, where pump flow rates and pressures are held constant, pulsatile flow demonstrably produces a significantly higher total hemodynamic energy and SHE compared with its nonpulsatile counterpart.^19,20^ This stark contrast highlights that both EEP and SHE are substantially elevated in pulsatile flow scenarios. Remarkably, SHE drops to zero under conditions of complete nonpulsatile flow, at which point EEP aligns directly with MAP. Figure 3 demonstrates the exceptional efficiency and remarkable advantages of pulsatile flow in enhancing hemodynamic performance.

## Commercially Available Pulsatile and Nonpulsatile Roller Pumps

After conducting a series of comprehensive experiments with the experimental University of Texas Neonate-Infant pulsatile pump, we decisively redirected our focus toward FDA-approved pulsatile roller pumps and a hydraulically driven nonocclusive two-chamber pulsatile pump.^17,18^ This innovative design features one chamber positioned before the oxygenator and another chamber after it, all tested rigorously using neonatal piglet models.^17^

In our investigation, we meticulously evaluated three distinct brands of pulsatile roller pumps alongside two brands of FDA-approved nonpulsatile systems. Additionally, we explored the capabilities of the hydraulically driven two-chamber physiologic pulsatile pump, which, while not currently FDA approved for neonates, demonstrated significant potential.^3^

Our results were striking: one of the roller pump systems with the pulsatile module failed to generate any degree of pulsatility.^3,20^ Conversely, the physiologic pulsatile pump produced dramatically higher SHE levels than its pulsatile and nonpulsatile counterparts in the neonatal piglet model (Supplemental Figure 2).^3^ Not only did it excel in performance but it also resulted in considerably less blood trauma compared to nonpulsatile roller pumps.^3,21^

### C) Translational Research and Clinical Trials at Penn State (2004-Present)

#### Advanced Circuit Optimization

**Figure 4.**
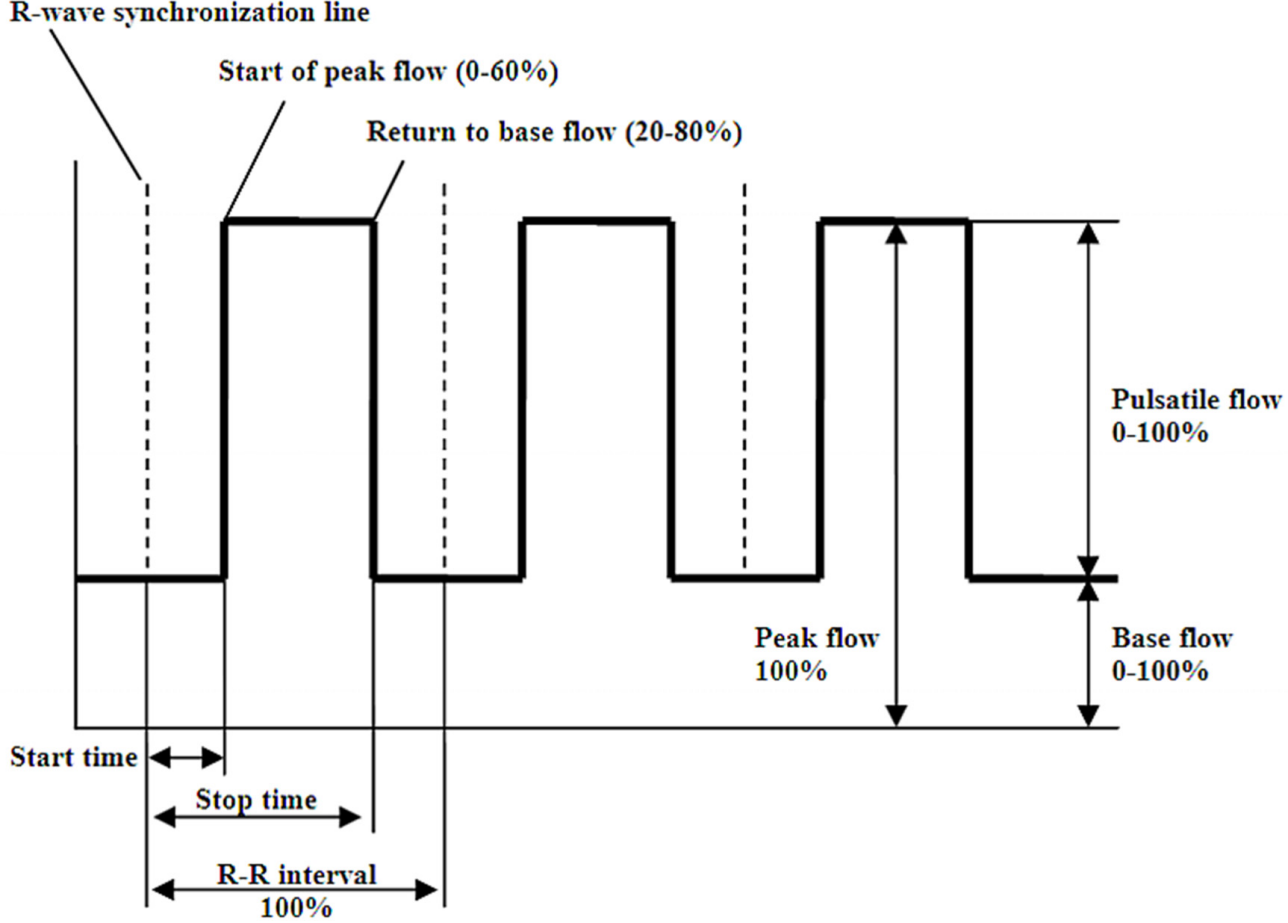
Pump settings diagram for pulsatile perfusion with a roller pump. [Reproduced with Permission from Rider AR, et al *ASAIO J*. 2009;55:100–105.].

We recognize the challenges associated with CPB circuit components that are not FDA-approved for clinical trials with pulsatile flow. This reality drives our commitment to robust, evidence-based translational research, which is essential for selecting circuit components that ensure the utmost safety when utilizing pulsatile flow. Our multidisciplinary research team has thoroughly evaluated an extensive array of FDA-approved hollow fiber membrane oxygenators, arterial filters, arterial cannulas, and appropriately sized arterial and venous lines.^22–27^ Our objective is to establish the safest circuit for all neonatal and pediatric CPB patients when pulsatile flow is preferred. Figure 4 diagrams these circuit pump settings for pulsatile perfusion with a roller pump.

Our approach combines rigorous hemodynamic evaluations with the latest advanced ultrasound technology, empowering us to precisely quantify gaseous microemboli (GME) counts and volumes across various oxygenator brands, perfusion modalities, and levels of vacuum-assisted venous drainage.^26–30^ This level of investigation is not just meticulous—it's necessary to drive meaningful improvements in patient care.

Moreover, we have identified concerning instances of diverted pump flows that can reach an alarming 40% to 50% of total flow, wherein blood is unintentionally rerouted back into the cardiotomy reservoir through a purge line of the arterial filter.^31^ This phenomenon, influenced by varying circuit pressures, pump flows, and tubing diameters, underscores the urgent need for continued rigorous research to safeguard our most vulnerable patients: neonates on CPB.

## Pilot Clinical Trials

To safely harness the advantages of pulsatile flow in pediatric patients, a pilot trial was conducted at Penn State Health Children's Hospital, enrolling 26 participants (13 patients in each group). This trial utilized transcranial Doppler ultrasound (TCD) to assess both pulsatile and nonpulsatile flows within the middle cerebral artery (MCA), focusing specifically on the pulsatility index as a key metric.^5^ The findings from this pilot trial demonstrated that the pulsatility index was significantly better preserved in the pulsatile group (0.8 during CPB compared to a baseline of 1.2) than in the nonpulsatile group (0.4 during CPB against a baseline of 1.2).

One of the most significant sources of morbidity following CPB is neurological injury. Our research center has conducted multiple pilot studies analyzing various proteins, their trends before, during, and after CPB, and their implications as biomarkers of neurological injury. One such study involved a cohort of 40 pediatric patients to determine how perfusion modality (n = 20 for each pulsatile and nonpulsatile perfusion) impacted proteins associated with the fibrinolytic system.^32^ Tissue plasminogen activator (tPA) and its inhibitor plasminogen activator inhibitor-1 (PAI-1) are proteins released by endothelial cells to help modulate physiological responses to white matter injury. A decreased level of PAI-1 is thought to predispose patients to tPA-induced injury. This study found a significant decrease of the PAI-1:tPA ratio for nonpulsatile patients 24-h post-CPB, indicating an imbalance in the fibrinolytic system that could predispose these patients to neurological injury.

A follow-up pilot study using the same patient population was conducted to investigate another biomarker, Apolipoprotein E (ApoE), a lipid-transport protein synthesized by neurons during periods of stress to aid in neuronal repair.^33^ Patients receiving pulsatile perfusion had lower levels of ApoE 24 h post-CPB compared with patients receiving nonpulsatile perfusion. Apolipoprotein E levels were also negatively correlated with cross-clamp time and CPB time for the nonpulsatile perfusion group. The results from this study imply that pulsatile perfusion may have neuroprotective properties. Additionally, when cross-clamp and CPB times, known predictors of more adverse outcomes, are prolonged with nonpulsatile flow, ApoE-induced neurological defense is impaired. These outcomes from pilot clinical trials emphasize the potential benefits of pulsatile flow in enhancing cerebral hemodynamics, and maintaining better ApoE levels, and the PAI-1:tPA ratio in pediatric patients while maintaining safety.

## A Primary Analysis of Clinical Outcomes from a Randomized Clinical Trial

Following circuit optimization, the next step from our research center sought to analyze the impact pulsatile and nonpulsatile perfusion modalities had on cerebral hemodynamics, vital organ injury, and short-term clinical outcomes in pediatric patients requiring surgery with CPB. In this randomized clinical trial, 159 patients were randomly assigned to either pulsatile or nonpulsatile perfusion.^6^ There were no differences in clinical outcomes between perfusion groups, including intubation time, intensive care unit (ICU) length of stay, and hospital length of stay. In the hours following CPB, both pulsatile and nonpulsatile groups showed progressive improvement in measures of vital organ injury as measured by PELOD-2 scores without any significant difference in score between the groups. The results of this primary analysis indicate that while pulsatile perfusion is a safe modality for CPB, it may not impart any improvement in clinical outcomes.

A secondary analysis from our randomized clinical trial^6^ was conducted to analyze the impact of perfusion modality on patients stratified by cyanotic or acyanotic heart disease.^8^ There was no difference in pulsatility index between cyanotic and acyanotic patients. PELOD-2 scores were similar between pulsatile and nonpulsatile perfusion patients among cyanotic and acyanotic cohorts. While PRISM-3 scores, a measure of mortality risk, were higher among all cyanotic patients compared with acyanotic patients, there were no differences in PRISM-3 scores within CHD groups between pulsatile and nonpulsatile patients. Other clinical outcomes, including intubation duration, ICU length of stay, and hospital length of stay, showed no differences between cyanotic versus acyanotic patients nor between pulsatile versus nonpulsatile patients within both CHD groups. Our findings from this secondary analysis reveal that even among cyanotic patients, who were shown to have longer aortic cross-clamp times, longer CPB times, and higher STAT mortality categories than acyanotic patients, there was no effect of perfusion modality on intubation times and ICU/hospital length of stays.

An additional analysis from this patient cohort was conducted to assess the differences in Total Tau levels between perfusion groups. In 144 of these patients, plasma samples were analyzed Total Tau, a protein biomarker linked with neurological injury. This analysis did not find any significant difference in Tau levels between perfusion modalities at any time in the perioperative period, up to 24 h post-CPB. The findings from this analysis and patient cohort once again imply that although it is safe, pulsatile perfusion may not improve the risk of adverse outcomes following surgery.^34^

A separate analysis from this patient cohort analyzed the differences in S100B levels between pulsatile and nonpulsatile perfusion patients. Similar to the Tau biomarker study, plasma samples from 144 patients were analyzed for S100B. This protein is released by glial cells in ischemic environments and thus could be a predictor of neurological injury. We found no significant differences in S100B levels between perfusion groups from before midline incision to 24 h post-CPB (*will be presented at the 62^nd^ Annual Meeting of the Society of Thoracic Surgeons, New Orleans, LA, January 29 – February 1, 2026).*

## Pulsatility Index and the Safety of Pulsatile Perfusion

**Figure 5.**
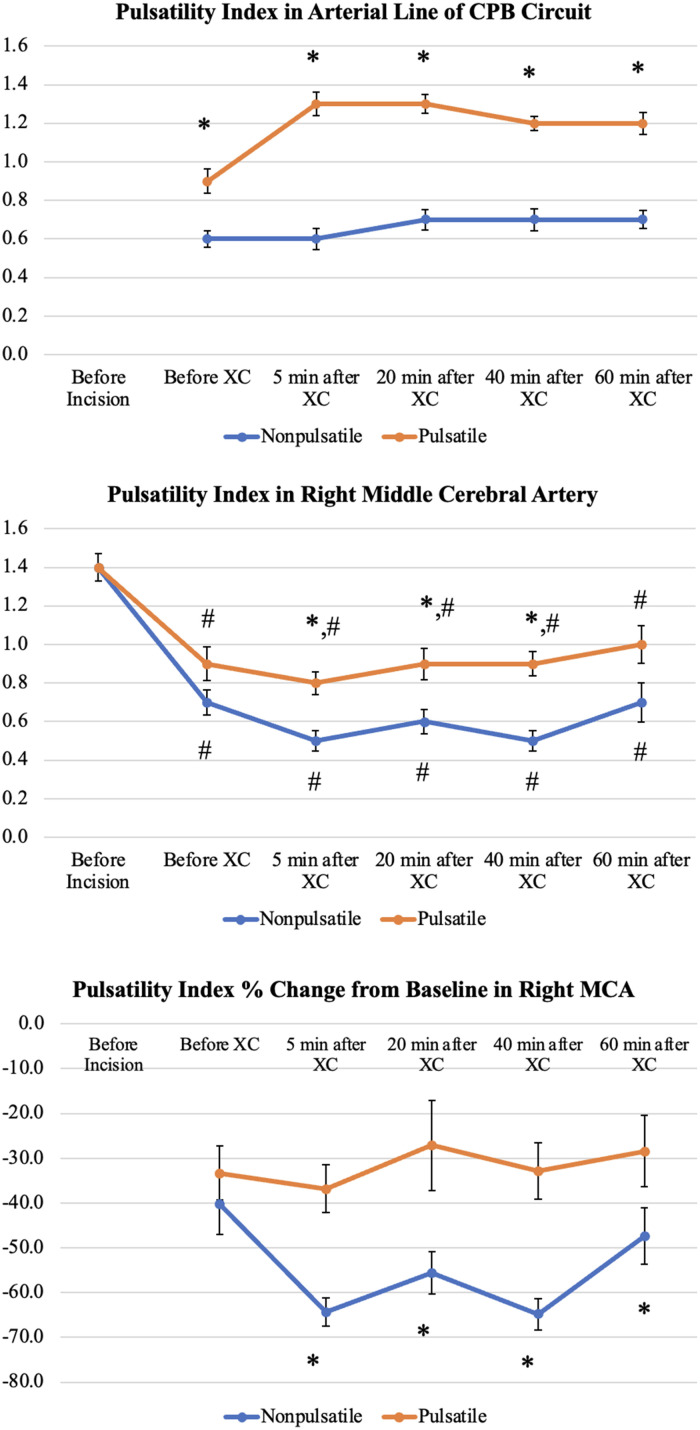
A comparison of the pulsatility index (PI) in three areas: the arterial line of the cardiopulmonary bypass (CPB) circuit (top), the right middle cerebral artery (middle), and the percentage change in PI from baseline in the right middle cerebral artery (MCA) between P and NP patients (bottom). *P < .05 between P versus NP groups (XC, cross-clamp). #*P* < .001 when comparing to baseline values (before incision) within the groups. [Reproduced with Permission from Ündar A, et al Ann Thorac Surg. 2022;114:1404–11].

It is important to note that, within these clinical trials, patients in the “nonpulsatile” group did experience a significant degree of pulsatility. Transcranial Doppler ultrasound waveforms showed pulsatile flow at the arterial line (Supplemental Figure 3). Based on our circuit optimization, it was not possible for truly “nonpulsatile” flow to be achieved. As a result, our experiments were comparing two pulsatile modalities, rather than strictly “pulsatile” versus “nonpulsatile.” Despite this, the pulsatility index was better maintained at the right MCA and in the arterial line in patients receiving pulsatile flow compared with those receiving nonpulsatile flow^6^ (Figure 5), indicating that pulsatile perfusion had an improved ability to mimic the physiological characteristics of a pulsatile circulatory system.

Our trials also indicated that pulsatile perfusion was a safe modality for CPB through measurements of plasma-free hemoglobin (PFH). In one of our first trials assessing the safety of pulsatile flow,^5^ PFH levels were rigorously measured both before and after CPB to evaluate hemolysis. This study found no significant differences in PFH between the pulsatile and nonpulsatile groups at any time (7.18 ± 1.15 mg/dL vs 9.08 ± 1.26 mg/dL, *P* = .30 before CPB; 40.91 ± 8.14 mg/dL vs 54.38 ± 8.9 mg/dL, *P* = .30 after CPB).^5^ Our trial assessing the impact of pulsatile versus nonpulsatile flow on cerebral hemodynamics found that pulsatile flow patients had a significantly lower PFH than nonpulsatile patients following CPB (40.6 ± 4.9 mg/dL vs 58.8 ± 7.3 mg/dL, *P* = .04).^6^

Gaseous microemboli counts were also measured in our clinical trials and retrospective studies to assess the safety of the different perfusion modalities. In our randomized trial,^6^ the number of emboli as measured by high-intensity transient signals at the MCA and arterial line were statistically similar. Similarly, a retrospective study found no difference in GME counts between pulsatility groups.^7^

Our findings from both clinical trials and our retrospective review reinforce the fact that though pulsatile perfusion can administer a pulsatility index closer to physiologic levels, it does not impart any improvement in short-term clinical outcomes compared to nonpulsatile perfusion. Regardless, as evident from our measurements of PFH and GME delivery, it remains a safe option to use for pediatric patients undergoing CPB.

### Retrospective Review

To further evaluate the effects of perfusion modality on cerebral hemodynamics and clinical outcomes, our research center conducted a retrospective review consisting of 284 total patients.^7^ Pulsatility index, GME counts, and mean flow velocity at the right MCA were measured via TCD, and near-infrared spectroscopy (NIRS) measured regional cerebral oxygen saturation (rSO_2_). Similar to our randomized clinical trial, pulsatility index was consistently higher in the pulsatile perfusion group compared with the nonpulsatile group at the arterial line and at the right MCA. There was no difference in GME counts or mean flow velocity between pulsatile and nonpulsatile perfusion groups. Additionally, although rSO_2_ decreased compared with baseline levels in both pulsatile and nonpulsatile perfusion modalities, there were no differences between the groups at any time before, during, and after CPB. With respect to clinical outcomes, pulsatile and nonpulsatile perfusion patients had similar short-term clinical outcomes, including intubation times, ICU/hospital length of stays, and mortality rates within 180 days.

When subdivided into low/middle-risk (STAT Mortality Categories 1-3) and high-risk (STAT Mortality Categories 4-5) groups, low/middle-risk patients receiving nonpulsatile perfusion had significantly shorter aortic cross-clamp times. In the high-risk category, nonpulsatile perfusion patients had significantly higher mortality scores, aortic cross-clamp times, CPB times, and longer hospital length of stays than pulsatile perfusion patients. It is worth considering that the higher baseline mortality scores among nonpulsatile patients may be the reason for the more adverse operative and clinical outcomes, rather than the perfusion modality. There were no differences in either risk group of GME delivery between pulsatile and nonpulsatile patients.

Our retrospective analysis revealed minimal differences in operative characteristics and clinical outcomes that could be attributed to perfusion modality. Other than pulsatility index, patients in both groups had similar cerebral hemodynamics during surgery and experienced similar outcomes afterwards. Despite our multidisciplinary research team's efforts to optimize the CPB circuit, our primary analysis and retrospective studies indicate that although it is a safe option, pulsatile perfusion is not a “magic bullet” that conveys added protection with respect to short-term clinical outcomes.^10^

## Regional Cerebral Oxygen Saturation

Another study from our research center was conducted to compare different measurements of cerebral hemodynamics between pulsatile (n = 77) and nonpulsatile (n = 34) perfusion patients using dual neuromonitoring strategies in TCD and NIRS.^35^ There were no differences in any demographic or perioperative characteristics between groups, including risk category, cross-clamp time, or CPB time. While both patient groups receiving pulsatile and nonpulsatile perfusion experienced decreases in pulsatility index compared with baseline, as measured by TCD, this decrease was greater in patients receiving nonpulsatile perfusion. Near-infrared spectroscopy data showed that patients receiving pulsatile perfusion experienced lower decreases in rSO_2_ levels compared with the nonpulsatile perfusion group throughout the surgery, which was statistically significant at 40 and 60 min following cross-clamp. Given that there were no differences between groups presurgery, the differences observed in pulsatility index and rSO_2_ may have been driven by perfusion modality. While it is unclear whether this difference is significant enough to determine clinical outcomes, any decrease in oxygen saturation could be a risk factor for adverse neurological outcomes. Again, pulsatile perfusion is shown to be a safe modality, and although it may not improve short-term clinical outcomes, it may help to reduce the risk of long-term neurological sequelae of CPB.

## Comment

After three decades of rigorous in vitro, in vivo, and clinical research comparing conventional nonpulsatile flow with alternative pulsatile flow in neonatal and pediatric congenital heart surgery patients, our multidisciplinary team has reached three essential conclusions:

1. *Optimization of the entire CPB circuitry:* It is imperative to optimize the entire CPB circuitry before initiating any clinical trial. This step is not merely a suggestion; it is a crucial necessity to ensure the safety and well-being of underserved congenital heart surgery patients worldwide. The establishment of custom-made pulsatile flow settings—tailoring pulse width, frequency, and more—on the roller pump, a careful selection of various brands of hollow fiber membrane oxygenators, and ensuring appropriate diameters for arterial and venous tubing and cannulas, is essential rather than optional. To date, no components of the CPB circuitry have received FDA approval, and device manufacturers have not provided evidence-based data to support the use of their products with pulsatile flow. Therefore, robust translational research is essential to optimize circuit components for those who favor pulsatile flow in clinical practice.
2. *Essential Quantification of Pulsatility*: The accurate and precise quantification of pulsatility is vital for making meaningful comparisons across studies from various institutions. Without a standardized approach, our progress and understanding in this field can be significantly impeded. While numerous hemodynamic energy formulas exist to quantify pressure and flow waveforms with precision, the pulsatility index obtained through TCD stands out as a well-established, real-time, and noninvasive method. This technique should be prioritized for the quantification of pulsatility in clinical practice to enhance our insights and foster advancements in medical research.
3. *Enhancing Patient Safety and Team Collaboration in Clinical Trials*: Engaging in comprehensive discussions about patient safety precautions with our multidisciplinary team is not just important; it is imperative before initiating any clinical trial. This collaborative effort is crucial to ensure the safety of our patients and uphold the integrity of our research. To safeguard our patients during procedures, it is essential to utilize a bubble detector in the arterial line of the CPB circuitry, along with an integrated arterial filter. Additionally, placing a separate flow probe after any shunts in the circuit—such as a purge line of the arterial filter—must be done proximal to the arterial cannula to accurately gauge the true pump flow to the patient. Given that smaller arterial circuits (less than 1/4 inch) can lead to significant increases in circuit pressures, up to 40% to 50% of the total pump flow can be diverted via the purge line of an arterial filter, particularly in neonatal patients. For this reason, employing a separate flow probe is essential. Furthermore, leveraging TCD technology is critical—not only for measuring the pulsatility index but also for detecting microemboli delivery in the MCA in real time and noninvasively. Utilizing TCD is strongly advised, particularly when pulsatile flow is preferred.

**Figure 6.**
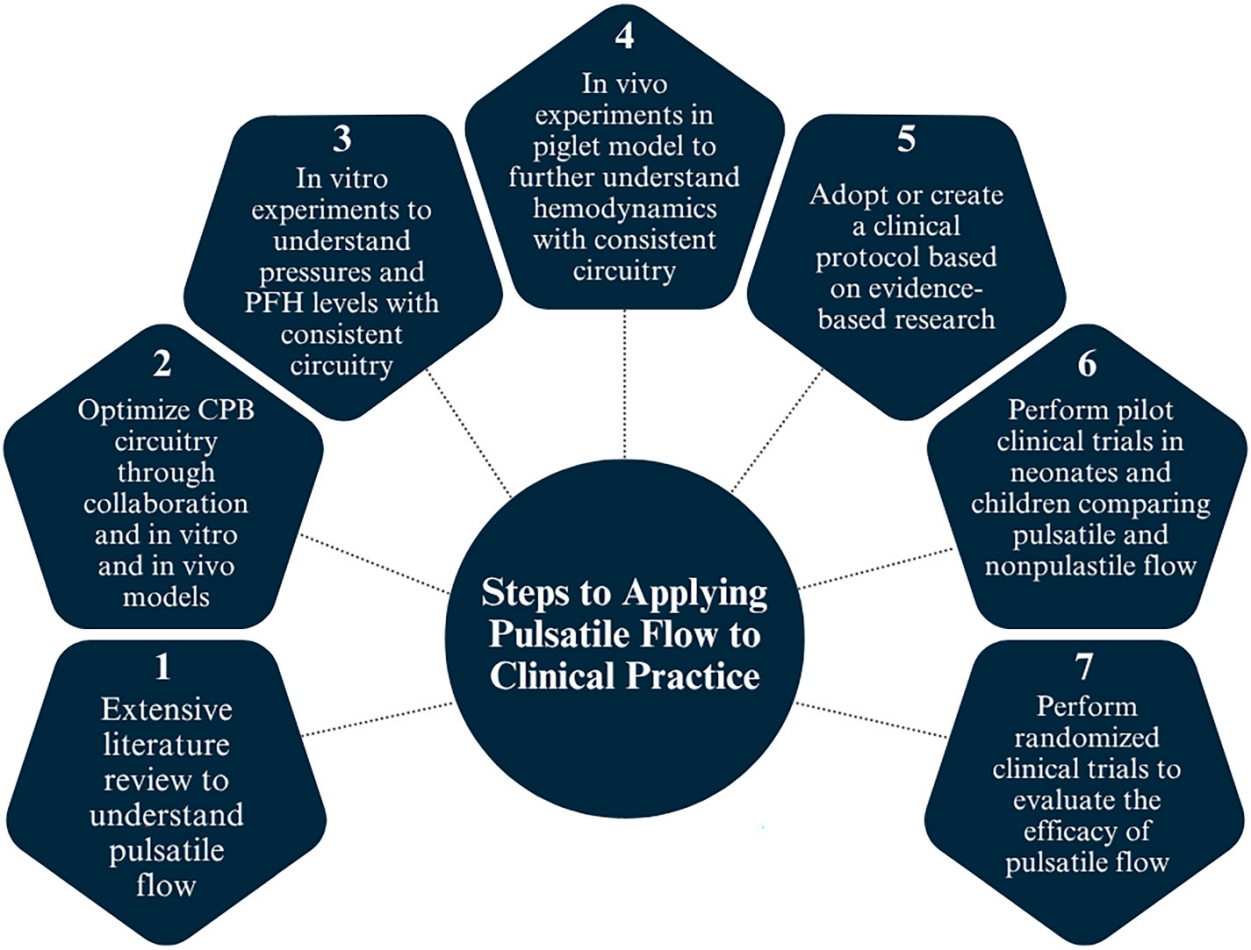
Steps to applying pulsatile flow to clinical practice. CPB, cardiopulmonary bypass; PFH, plasma-free hemoglobin.

Incorporating pulsatile flow in clinical practice not only improves the pulsatility index at the MCA but also adds complexity to the CPB circuitry. Therefore, it is vital to implement additional safety measures, as outlined above, alongside a strategic seven-step approach to ensure optimal patient safety and procedural success (Figure 6).

## Conclusion

After dedicating the past three decades to a wide range of translational, engineering, basic science, and clinical research projects, we have come to the realization that there is no clear evidence that pulsatile CPB offers greater short-term or long-term benefits for our pediatric patients undergoing congenital heart surgery. We appreciate the insights gained from pilot clinical trials, which have shown some promising improvements in ApoE and PAI-1/tPA ratios. However, our comprehensive randomized clinical trial involving 159 patients, along with a thorough retrospective study of 284 patients at Penn State Health Children's Hospital, did not demonstrate any significant clinical benefit of pulsatile flow compared with nonpulsatile flow. It is important to note that, although pulsatile flow did not show measurable benefits, it did not lead to any adverse outcomes in our clinical trials. It is crucial to emphasize that during the clinical trials conducted at Penn State Health Children's Hospital, patients classified within the “nonpulsatile” group did, in fact, experience a noteworthy degree of pulsatility. Transcranial Doppler ultrasound waveforms revealed clear pulsatile flow at the arterial line (see Supplemental Figure 3 and Figure 5). Due to our circuit optimization efforts, achieving a truly “nonpulsatile” flow proved to be impossible. Consequently, our experiments were effectively comparing two pulsatile modalities, rather than a strict dichotomy of “pulsatile” versus “nonpulsatile.” This insight underscores the complexity of our findings and highlights the need for a nuanced understanding of the results.

In summary, pulsatile flow is not a panacea. It should only be considered after completing all necessary in-vitro and in-vivo studies using a consistent clinical CPB setup for neonatal and pediatric patients. Skipping these critical steps could not only fail to improve clinical outcomes but may also introduce significant safety risks for this vulnerable patient population.

## Supplemental Material

sj-docx-1-pch-10.1177_21501351251412887 - Supplemental material for Three Decades of Evidence on Pulsatile Cardiopulmonary Bypass in Pediatrics: No Significant Clinical Benefit Supplemental material, sj-docx-1-pch-10.1177_21501351251412887 for Three Decades of Evidence on Pulsatile Cardiopulmonary Bypass in Pediatrics: No Significant Clinical Benefit by Akif Ündar, Marc J. Lussier, Srihari Rajesh and Kevsergül Dayi in World Journal for Pediatric and Congenital Heart Surgery

sj-tiff-2-pch-10.1177_21501351251412887 - Supplemental material for Three Decades of Evidence on Pulsatile Cardiopulmonary Bypass in Pediatrics: No Significant Clinical Benefit Supplemental material, sj-tiff-2-pch-10.1177_21501351251412887 for Three Decades of Evidence on Pulsatile Cardiopulmonary Bypass in Pediatrics: No Significant Clinical Benefit by Akif Ündar, Marc J. Lussier, Srihari Rajesh and Kevsergül Dayi in World Journal for Pediatric and Congenital Heart Surgery

sj-tiff-3-pch-10.1177_21501351251412887 - Supplemental material for Three Decades of Evidence on Pulsatile Cardiopulmonary Bypass in Pediatrics: No Significant Clinical Benefit Supplemental material, sj-tiff-3-pch-10.1177_21501351251412887 for Three Decades of Evidence on Pulsatile Cardiopulmonary Bypass in Pediatrics: No Significant Clinical Benefit by Akif Ündar, Marc J. Lussier, Srihari Rajesh and Kevsergül Dayi in World Journal for Pediatric and Congenital Heart Surgery

sj-tiff-4-pch-10.1177_21501351251412887 - Supplemental material for Three Decades of Evidence on Pulsatile Cardiopulmonary Bypass in Pediatrics: No Significant Clinical Benefit Supplemental material, sj-tiff-4-pch-10.1177_21501351251412887 for Three Decades of Evidence on Pulsatile Cardiopulmonary Bypass in Pediatrics: No Significant Clinical Benefit by Akif Ündar, Marc J. Lussier, Srihari Rajesh and Kevsergül Dayi in World Journal for Pediatric and Congenital Heart Surgery

## Abbreviations

ApoE: Apolipoprotein E
CBF: cerebral blood flow
CHD: congenital heart disease
CPB: cardiopulmonary bypass
CPP: cerebral perfusion pressure
DHCA: deep hypothermic circulatory arrest
EEP: energy equivalent pressure
GME: gaseous microemboli
MAP: mean arterial pressure
MCA: middle cerebral artery
PAI-1: plasminogen activator inhibitor-1
PELOD-2: Pediatric Logistic Organ Dysfunction-2
PRISM-3: Pediatric Risk of Mortality 3
rSO_2_: regional cerebral oxygen saturation
SHE: surplus hemodynamic energy
TCD: transcranial doppler ultrasound
tPA: tissue plasminogen activator
VIS: Vasoactive–Inotropic Score
