# Supplementary material for: Three Decades of Evidence on Pulsatile Cardiopulmonary Bypass in Pediatrics: No Significant Clinical Benefit
Source: World J Pediatr Congenit Heart Surg. 2026 Feb 4;17(5):655–65. doi: 10.1177/21501351251412887 (PMC13443370; doi:10.1177/21501351251412887)
Supplement: Supplementary file 1 [file sj-docx-1-pch-10.1177_21501351251412887.docx]

**Supplemental Table 1. In vitro and Neonatal Piglet Studies on the University of Texas Neonate-Infant Displacement Pump**

| Year | Article | Main Findings |
| --- | --- | --- |
| 1996 | Ündar A. Design and Performance of Physiologic Pulsatile Flow Cardiopulmonary Bypass Systems for Neonates and Infants. PhD Dissertation, The University of Texas at Austin, Biomedical Engineering, Austin, Texas, pp. 1-343, 1996. | This was the initial design and extensive evaluation of the University of Texas Neonatal Pulsatile pump, showing that it could produce a phyiologic form a pulsatile flow. |
| 1996 | Ündar A, Runge TM, Miller OL, et al. Design of a Physiologic Pulsatile Flow Cardiopulmonary Bypass System for Neonates and Infants. Int J Artif Organs. 1996;19:170-176. | The University of Texas Neonatal Pulsatile pump produced a physiological blood flow waveform. With varying motor speeds, different physiological pressure waveforms, pump rates, and stroke volumes were produced. |
| 1996 | Ündar A, Lodge AJ, Runge TM, Daggett CW, Ungerleider RM, Calhoon JH. Design and performance of a physiologic pulsatile flow neonate-infant cardiopulmonary bypass system. *ASAIO J*. 1996;42:M580-M583. | UT Austin Pump produces physiologic pulsatile flow and matches the target stroke volume for neonates. |
| 1997 | Lodge AJ, Ündar A, Daggett CW, Runge TM, Calhoon JH, Ungerleider RM. Regional blood flow during pulsatile cardiopulmonary bypass and after circulatory arrest in an infant model. *Ann Thorac Surg.* 1997;63:1243-1250. | When compared with a standard nonpulsatile roller pump, the UT Austin pump maintained myocardial blood flow at or above baseline throughout the study, with no significant differences in cerebral blood flow. |
| 1998 | Ündar A, Lodge AJ, Daggett CW, Runge TM, Ungerleider RM, Calhoon JH. The type of aortic cannula and membrane oxygenator affect the pulsatile waveform morphology produced by a neonate-infant cardiopulmonary bypass system in vivo. *Artif Organs*. 1998;22:681-686. | UT Austin Pump produces the best physiologic pulsatile flow when paired with a 10 Fr aortic cannula and the Capiox 308 hollow fiber membrane oxygenator. |
| 1998 | Ündar A, Holland MC, Howelton RV, et al. Testing neonate-infant membrane oxygenators with the University of Texas neonatal pulsatile cardiopulmonary bypass system in vitro. Perfusion. 1998;13:346-352. | The Capiox 308 oxygenator produced the greatest upstroke and lowest ejection time and extracorporeal circuit pressure with pulsatile flow when compared with the Minimax Plus, Masterflo Infant, and VPCML Plus. |
| 2000 | Ündar A, Vaughn WK, Calhoon JH. The effects of cardiopulmonary bypass and deep hypothermic circulatory arrest on blood viscoelasticity and cerebral blood flow in a neonatal piglet model. *Perfusion*. 2000;15:121-128. | Regional blood flow and viscoelasticity diminished after 60 min of DHCA, and it did not return to pre-CPB levels even after 60 min post-CPB. Pulsatile flow does not hinder the effects of DHCA. |
| 2001 | Ündar A, Vaughn WK, Calhoon JH: Effects of pulsatile versus nonpulsatile flow on cerebral hemodynamics during pediatric cardiopulmonary bypass with deep hypothermic circulatory arrest. *Proc IEEE Eng Med Biol* 2001;23:0–4. | The UT Austin Pump produced higher cerebral blood flow, higher cerebral metabolic rate of oxygen, and higher cerebral oxygen delivery than the nonpulsatile roller pump. |

**Supplemental Table 2. In vitro and Neonatal Piglet Studies with the Experimental Physiologic Pulsatile Pump and Other Commercially Available Pulsatile and Nonpulsatile Roller Pumps**

| Year | Article | Main Findings |
| --- | --- | --- |
| 1999 | Ündar A, Masai T, Inman R, et al. Evaluation of a physiologic pulsatile pump system for neonate-infant cardiopulmonary bypass support. *ASAIO J.* 1999;45:53-58. | The hydraulically driven physiologic pulsatile pump with a 10 French DLP aortic cannula and an arterial filter produced adequate pulsatility. |
| 1999 | Ündar A, Frazier OH, Fraser Jr. CD. Defining Pulsatile Perfusion: Quantification in Terms of Energy Equivalent Pressure. Artif Organs. 1999;23:712-716. | Pressure flow waveforms with both pulsatile and nonpulsatile perfusion can be precisely quantified with energy equivalent pressure (EEP). |
| 1999 | Ündar A, Masai T, Yang SQ, Goddard-Finegold J, Frazier OH, Fraser CD Jr. Effects of perfusion mode on regional and global organ blood flow in a neonatal piglet model. *Ann Thorac Surg*. 1999;68:1336-1343. | Cerebral, renal, and myocardial blood flow was better maintained with the pulsatile roller pump than nonpulsatile roller pump. |
| 1999 | Ündar A, Henderson N, Thurston GB, et al. The effects of pulsatile versus nonpulsatile perfusion on blood viscoelasticity before and after deep hypothermic circulatory arrest in a neonatal piglet model. *Artif Organs*. 1999;23:717-721. | The experimental pulsatile pump produced less blood trauma than the standard nonpulsatile roller pump since the pulsatile pump produced lower levels of viscoelasticity. |
| 1999 | Ündar A, Masai T, Frazier OH, Fraser CD Jr. Pulsatile and nonpulsatile flows can be quantified in terms of energy equivalent pressure during cardiopulmonary bypass for direct comparisons. *ASAIO J.* 1999;45:610-614. | The experimental pulsatile pump generates greater hemodynamic energy and better regional blood flow with reduced vascular resistance than pulsatile and nonpulsatile roller pumps |
| 2000 | Ündar A, Eichstaedt HC, Frazier OH, Fraser CD Jr. Monitoring regional cerebral oxygen saturation using near-infrared spectroscopy during pulsatile hypothermic cardiopulmonary bypass in a neonatal piglet model. *ASAIO J.* 2000;46:103-106. | This commercially available pulsatile roller pump is a reliable pulsatile device and generates higher energy than nonpulsatile pumps. There was no cerebral oxygen saturation deficiency in this particular pulsatile model during CPB. |
| 2000 | Ündar A, Koenig KM, Frazier OH, Fraser CD. Impact of membrane oxygenators on pulsatile versus nonpulsatile perfusion in a neonatal model. Perfusion. 2000;15:111-120. | The Capiox SX10 produced lower ECP and pressure drops compared to the Lilliput 901 when tested with the Jostra HL-20 roller pump and with pulsatile and nonpulsatile perfusion. |
| 2001 | Ündar A, Eichstaedt HC, Masai T, et al. Comparison of six pediatric cardiopulmonary bypass pumps during pulsatile and nonpulsatile perfusion. *J Thorac Cardiovasc Surg.* 2001;122:827-829. | The experimental pulsatile pump produced the highest hemodynamic energy than all other pumps. The pulsatile roller pumps produced a significantly higher average increase in hemodynamic energy than the nonpulsatile pumps. |
| 2001 | Ündar A, Masai T, Yang SQ, et al. Global and regional cerebral blood flow in neonatal piglets undergoing pulsatile cardiopulmonary bypass with continuous perfusion at 25 degrees C and circulatory arrest at 18 degrees C. *Perfusion*. 2001;16:503-510. | Both deep hypothermic circulatory arrest (DHCA) and hypothermic cardiopulmonary bypass significantly reduced regional blood flow during CPB using the PPP, but DHCA continued to impair flow after rewarming. |
| 2002 | Ündar A, Masai T, Beyer EA, Goddard-Finegold J, McGarry MC, Fraser CD Jr. Pediatric physiologic pulsatile pump enhances cerebral and renal blood flow during and after cardiopulmonary bypass. *Artif Organs.* 2002;26:919-923. | Pulsatile flow may minimize cerebral injury after DHCA because pulsatile flow generates higher hemodynamic energy, improving cerebral and regional blood flow during and after CPB with DHCA. |
| 2002 | Ündar A, Masai T, Yang SQ, et al. Pulsatile perfusion improves regional myocardial blood flow during and after hypothermic cardiopulmonary bypass in a neonatal piglet model. *ASAIO* *J*. 2002;48:90-95. | Pulsatile perfusion using the experimental pulsatile pump improved myocardial blood flow after hypothermic CPB in comparison to CPB with a nonpulsatile roller pump. |
| 2005 | Ündar A, Eichstaedt HC, Masai T, Bigley JE, Kunselman AR. Precise quantification of pulsatility is a necessity for direct comparisons of six different pediatric heart-lung machines in a neonatal CPB model. *ASAIO J*. 2005;51:600-603. | The experimental pulsatile pump produced the greatest surplus hemodynamic energy whereas the nonpulsatile pumps and one pulsatile roller pump did not generate any surplus hemodynamic energy. |

**Supplemental Table 3. In vitro Studies of Commercially Available CPB Components and Clinical Studies Evaluating Pulsatile Flow at Penn State**

| Year | Article | Main Findings |
| --- | --- | --- |
| 2006 | Ündar A, Ji B, Lukic B, et al. Quantification of Perfusion Modes in Terms of Surplus Hemodynamic Energy Levels in a Simulated Pediatric CPB Model. ASAIO J. 2006;52:712-717. | Pulsatile perfusion produced greater SHE levels compared to nonpulsatile perfusion. |
| 2006 | Ündar A, Ji B, Lukic B, et al. Comparison of hollow-fiber membrane oxygenators with different perfusion modes during normothermic and hypothermic CPB in a simulated neonatal model. Perfusion. 2006;21:381-390. | The Capiox Baby RX produced lower pressure drops compared to the Lilliput 1-D901, and pulsatile flow produced greater SHE levels with either oxygenator compared to nonpulsatile flow. |
| 2007 | Ündar A, Ji B, Kunselman AR, Myers JL. Detection and Classification of Gaseous Microemboli During Pulsatile and Nonpulsatile Perfusion in a Simulated Neonatal CPB Model. ASAIO J. 2007;53:725-729. | Pulsatile flow created and delivered more GME to the post pump site, but no significant differences in GME counts were shown after the oxygenator. |
| 2007 | Ündar A, Ji B, Rider A, et al. Comparison of Four Different Pediatric 10F Aortic Cannulae During Pulsatile Versus Nonpulsatile Perfusion in a Simulated Neonatal Model of Cardiopulmonary Bypass. ASAIO J. 2007;53:778-784. | Pulsatile flow yielded greater pressures, and pressure drops. Pulsatile flow provided greater SHE levels with all cannulas. When assessing each cannula, the Surgimedics cannula created the greatest pressure drops and lowest SHE levels. |
| 2008 | Rider AR, Ji B, Kunselman AR, Weiss WJ, Myers JL, Ündar A. A Performance Evaluation of Eight Geometrically Different 10 Fr Pediatric Arterial Cannulae Under Pulsatile and Nonpulsatile Perfusion Conditions in an Infant Cardiopulmonary Bypass Model. ASAIO J. 2008;54:306-315. | Pulsatile perfusion yielded greater circuit pressures, pressure drops, and SHE levels. The Surgimedics and THI cannulas had the greatest pressure drops and lowest SHE levels. |
| 2008 | Schreiner RS, Rider AR, Myers JW, et al. Microemboli Detection and Classification by Innovative Ultrasound Technology During Simulated Neonatal Cardiopulmonary Bypass at Different Flow Rates, Perfusion Modes, and Perfusate Temperatures. ASAIO J. 2008;54:316-324. | Like previous experiments, pulsatile flow created and delivered more GME to the post pump site. Greater flow rates and lower temperatures also generated greater GME counts. |
| 2008 | Wang S, Baer L, Kunselman AR, Myers JL, Ündar A. Delivery of Gaseous Microemboli With Vacuum-Assisted Venous Drainage During Pulsatile and Nonpulsatile Perfusion in a Simulated Neonatal Cardiopulmonary Bypass Model. ASAIO J. 2008;54:416-422. | GME counts by perfusion modality were similar to previous experiments, but higher negative pressures with VAVD created and delivered more GME post pump. |
| 2008 | Wang S, Miller A, Myers JL, Undar A. “Stolen” Blood Flow: Effect of an Open Arterial Filter Purge Line in a Simulated Neonatal CPB Model. ASAIO J. 2008;54:432-435. | Purge lines have been shown to divert and shunt blood flow to the patients in both pulsatile and nonpulsatile flow which creates the need for further evaluation and measurement of flow rates. |
| 2008 | Wang S, Win KN, Kunselman AR, Woitas K, Myers JL, Ündar A. The Capability of Trapping Gaseous Microemboli of Two Pediatric Arterial Filters With Pulsatile and NonPulsatile Flow in a Simulated Infant CPB Model. ASAIO J. 2008;54:519-522. | When comparing the Medtronic Affinity and Terumo Capiox arterial filters, both generated similar results in terms of pressure drops and GME capturing. But higher flow rates and pulsatile perfusion created higher GME counts. |
| 2008 | Wang S, Kunselman AR, Myers JL, Ündar A. Comparison of Two Different Blood Pumps on Delivery of Gaseous Microemboli During Pulsatile and NonPulsatile Perfusion in a Simulated Infant CPB Model. ASAIO J. 2008;54:538-541. | The Medos deltrastream DP1 delivered fewer GME to the post pump site compared to the Jostra roller pump, but most GME were trapped in the oxygenator and there were very few at the postfilter site. |
| 2009 | Wang S, Haines N, Ündar A. Hemodynamic Energy Delivery of the Pulsatile Flow in a Simulated Pediatric Extracorporeal Circuit. ASAIO J. 2009;55:96-99. | When measuring pressures and THE levels with pulsatile and nonpulsatile perfusion, pressure, SHE, and THE levels were shown to drop at each measured circuit component but pulsatile flow delivered the greatest THE levels |
| 2009 | Rider AR, Ressler NM, Karkhanis TR, Kunselman AR, Wang S, Ündar A. The Impact of Pump Settings on the Quality of Pulsatility. ASAIO J. 2009;55:100-105. | Lower baseflows resulted in higher MAP, EEP, SHE, and THE. Earlier pump head start times resulted in lower MAP, and higher EEP, SHE, and THE. From this it was understood that lower baseflows and smaller durations between pump start and stop produced the best pulsatility in terms of SHE and THE. |
| 2009 | Wang S, Haines N, Ündar A. Quantification of pressure-flow waveforms and selection of components for the pulsatile extracorporeal circuit. J Extra Corpor Technol. 2009;41:20-25. | In addition to EEP, pressure flow waveforms can also be precisely quantified with total hemodynamic energy (THE) and surplus hemodynamic energy (SHE). Comparisons of different components done previously in terms of these values were summarized. |
| 2009 | Haines NM, Wang S, Kunselman A, Myers JL, Ündar A. Comparison of Pumps and Oxygenators With Pulsatile and Nonpulsatile Modes in an Infant Cardiopulmonary Bypass Model. Artif Organs. 2009;33:993-1001. | The Jostra roller pump and Medos deltrastream DP1 were evaluated with the Capiox RX05 and Medos HiLite 800LT oxygenators. The Jostra pump produced better pulsatile flow than the Medos pump. The Capiox oxygenator had greater pressure drops with the Medos pump at particular flow rates when compared with the Medos oxygenator. |
| 2010 | Rogerson A, Guan Y, Kimatian SJ, Kunselman AR, Clark JB, Myers JL, et al. Transcranial Doppler ultrasonography: A reliable method of monitoring pulsatile flow during cardiopulmonary bypass in infants and young children. J Thorac Cardiovasc Surg. 2010;139:e80-2. | Transcaranial doppler was used to measure Gosling’s pulsatility index (PI) in 26 pediatric patients undergoing either pulsatile or nonpulsatile flow. PI was greater in the pulsatile group and mean arterial pressure was greater in the pulsatile group. |
| 2011 | Su XW, Guan Y, Barnes M, Clark JB, Myers JL, Ündar A. Improved Cerebral Oxygen Saturation and Blood Flow Pulsatility With Pulsatile Perfusion During Pediatric Cardiopulmonary Bypass. Pediatr Res. 2011;70:181-5. | Cerebral oxygen saturation (rSO_2_) and pulsatility index (PI) was measured in 111 patients randomized between pulsatile flow and nonpulsatile flow. The pulsatile group showed a lower decrease in rSO_2_ and PI compared to the nonpulsatile group. |
| 2014 | Aĝirbaşli MA, Song J, Lei F, Wang S, Kunselman AR, Clark JB, et al. Comparative Effects of Pulsatile and Nonpulsatile Flow on Plasma Fibrinolytic Balance in Pediatric Patients Undergoing Cardiopulmonary Bypass. Artif Organs. 2014;38:28-33. | 40 children (20 pulsatile, 20 nonpulsatile) were measured for tissue plasminogen activator (tPA) and plasminogen activator inhibitor-1 (PAI-1). The results showed that pulsatile flow maintained endogenous fibrinolytic balance. |
| 2015 | Aĝirbaşli MA, Song J, Lei F, Wang S, Kunselman AR, Clark JB, et al. Apolipoprotein E Levels in Pediatric Patients Undergoing Cardiopulmonary Bypass. Artif Organs. 2015;39:28-33. | Apolipoprotein E (apoE) levels were assessed with pulsatile and nonpulsatile flow in 38 pediatric patients. ApoE could play a critical role in modulating the response to neurological injury. Pulsatile flow was associated with lower apoE levels ApoE levels also negatively correlated with cross clamp time and pump time in the nonpulsatile group. |
| 2018 | Moroi M, Force M, Wang S, Kunselman AR, Ündar A. In Vitro Comparison of Pediatric Oxygenators With and Without Integrated Arterial Filters in Maintaining Optimal Hemodynamic Stability and Managing Gaseous Microemboli. Artif Organs. 2018;42:420-431. | The Capiox FX15 (with integrated arterial filter) and Capiox RX15 (with separate Capiox AF125 arterial filter) performed similarly hemodynamically, but pulsatile flow produced greater SHE and THE levels. GME were removed by both oxygenators at lower flow rates. Pulsatile flows at 4 L/min generated instantaneous flow rates that exceeded the documented maximum limits of the oxygenators, likely facilitating the delivery of GME to the pseudo-patient. |
| 2022 | Ündar A, Patel K, Holcomb RM, Clark JB, Ceneviva GD, Young CA, et al. A Randomized Clinical Trial of Perfusion Modalities in Pediatric Congenital Heart Surgery Patients. Ann Thorac Surg. 2022;114:1404-11. | In this clinical trial pulsatile flow produced a more physiologic flow pattern compared with nonpulsatile flow but showed no difference in clinical outcomes or vital organ injury. |
| 2023 | Patel K, Dan Y, Kunselman AR, Clark JB, Myers JL, Ündar A. The effects of pulsatile versus nonpulsatile flow on cerebral pulsatility index, mean flow velocity at the middle cerebral artery, regional cerebral oxygen saturation, cerebral gaseous microemboli counts, and short-term clinical outcomes in patients undergoing congenital heart surgery. JTCVS Open. 2023;16:786-800. | In this retrospective study of a patient cohort, pulsatile flow was shown to only have a greater pulsatility index than nonpulsatile flow. Short term clinical outcomes and cerebral perfusion were similar between pulsatile and nonpulsatile groups. |
| 2025 | Patel K, Lin TK, Clark JB, Ceneviva GD, Imundo JR, Spear D, et al. Randomized Trial of Pulsatile and Nonpulsatile Flow in Cyanotic and Acyanotic Congenital Heart Surgery. World J Pediatr Congenit Heart Surg. 2025;16:329-37. | This clinical trial compared pulsatile and nonpulsatile flow in cyanotic and acyanotic CHD. No clinical differences were found within acyanotic and cyanotic groups between pulsatile and nonpulsatile flow. |
| 2025 | Hayes CC, Ündar A. Clinical Assessment of the Utility of Pulsatile Over Nonpulsatile Flow for Neonatal and Pediatric Cardiopulmonary Bypass Procedures Using Multidisciplinary Translational Research at a Tertiary Care Center. World J Pediatr Congenit Heart Surg. 2025;16:354-356. | When studying pulsatile flow, it is important to adopt a multidisciplinary, translational approach. This should start with literature reviews, then move towards in vitro and in vivo studies. These should work toward a clinical protocol, pilot clinical trials, and randomized clinical trials. |

**Supplemental Figure Legends**

**Supplemental Figure 1**. **Pressure and flow waveforms in neonatal/pediatric cardiopulmonary bypass circuit proximal to the 10F aortic cannula in vitro using the University of Texas Neonatal Pulsatile Pump.**

[Reproduced with permission Ündar A, *et al.* *Int J Artif Organs.* 1996;19:170-176].

**Supplemental Figure 2.** **Levels of surplus hemodynamic energy in neonatal piglets using six different heart-lung machines during normothermic cardiopulmonary bypass**. [PPP = Physiologic Pulsatile Pump; Jostra-PR = Jostra Pulsatile Roller; SIII-PR = Stockert SIII Pulsatile Roller; SIII-Mast-PR = Stockert mast-mounted pulsatile roller; SIII-Mast-NP = Stockert SIII mast-mounted non-pulsatile roller; CAPS-NP = Stockert CAPS nonpulsatile roller pump]

[Reproduced with permission from Ündar A, *et al.* *ASAIO J*. 2005;51:600-603.

**Supplemental Figure 3**. **Blood flow waveforms for right middle cerebral artery (MCA) and the arterial line acquired using transcranial doppler (TCD) at 3 time points from A) pulsatile and B) non-pulsatile cohorts.** Patient weight: 14.4 for pulsatile cohort and 13.1 kg for non-pulsatile cohort. (CPB, cardiopulmonary bypass).

[Reproduced with permission from Ündar A, et al. *Ann Thorac Surg*. 2022;114:1404-11].
